# Coronary Artery and Pulmonary Artery Fistula Originated from Significant Stenosis in the Left Anterior Descending Artery

**DOI:** 10.1155/2013/298156

**Published:** 2013-02-12

**Authors:** Alper Sami Kunt

**Affiliations:** Cardiovascular Surgery Clinic, Yasam Hastanesi, 1486 Sokak, Muratpasa, Antalya, Turkey

## Abstract

Coronary artery fistula (CAF) is defined as a rare anomalous connection between a coronary artery and a major vessel or a cardiac chamber. We report a case of a left anterior descending coronary (LAD) stenosis and coronary artery fistula between the LAD coronary artery and the pulmonary artery (PA). CAF is often diagnosed by coronary angiogram. We describe our diagnostic approach and review the literature on the epidemiology, the pathophysiology, the diagnostic modalities, and the treatment options.

## 1. Background

Coronary artery fistulas between the LAD and the PA are rare congenital malformations; however, concomitant significant coronary artery stenosis can cause coronary steal phenomenon and this results in myocardial ischemia [1].

## 2. Case Presentation

**Figure 1:**
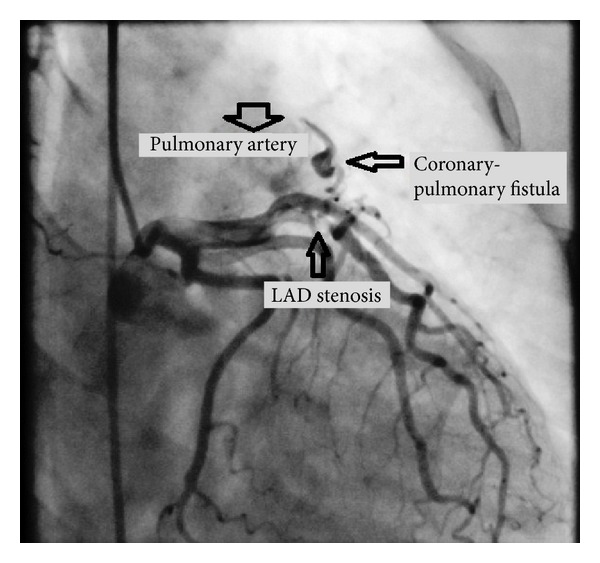
Angiographic view of coronary fistula between left internal thoracic artery and pulmonary artery.

**Figure 2:**
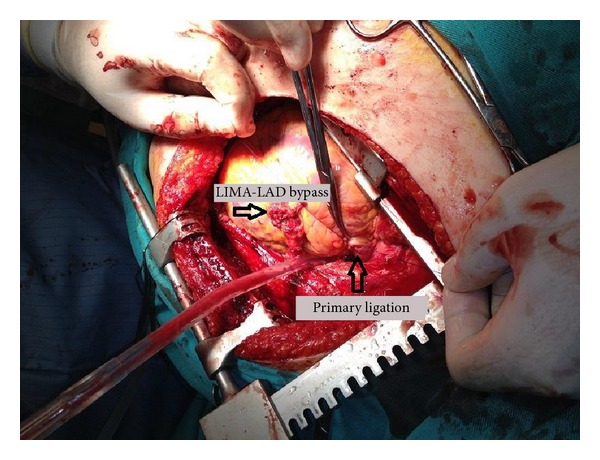
Coronary bypass and fistula ligation.

A 64-year-old female was admitted at our hospital for management of unstable angina. Coronary angiography revealed severe atherosclerotic coronary artery disease associated with an LAD-PA fistula (Figure 1). Coronary artery catheterization showed proximal stenosis in the left anterior descending coronary artery. Physical examination revealed continuous murmur. Ejection fraction was measured as 65%. After providing informed consent, the patient underwent coronary artery bypass grafting through a sternotomy in May 2011, without cardiopulmonary bypass. Left internal thoracic artery was anastomosed to the left anterior descending coronary artery. After identification of the LAD-PA fistula, it was sutured on both sides with 4/0 polypropylene (Figure 2). Special attention was directed to the absence of thrill, which may suggest the possibility of residual fistula, and the thrill was disappeared manually. The patient had an uneventful postoperative course and was discharged on postoperative day 6 in good clinical condition. During 11 months of followup, the patient had an excellent quality of life without subsequent cardiac events.

## 3. Conclusions

Fistula between coronary artery and pulmonary artery is a rare congenital anomaly. It was first described by Krause in 1865, but the first successful surgical treatment was described by Fell and colleagues [2] in 1958. 

Coronary artery fistula causes myocardial ischemia both by producing a coronary steal and by imposing an additional volume load on the left ventricle. However, these patients with coronary artery fistula are entirely asymptomatic [1, 3–5]. If the fistula is associated with atherosclerotic coronary artery disease, angina pectoris may be one of the symptoms and although it is rare, myocardial infarction may also develop. Our patient had both severe coronary artery disease and coronary fistula those aggravates each other's clinic. 

There is no specific medical therapy for coronary fistula, yet in some cases the fistula can be treated by transcatheter embolization or surgical intervention [6–8]. In our patient, the LAD-PA fistula was an incidental diagnosis associated with the significant coronary artery disease, and both conditions were surgically treated. The main goal was to perform coronary artery bypass grafting and then close the fistula through both sides with ligation during the same procedure while heart beating. The operation was completed by the favorable postoperative course. 

In conclusion, surgery with or without cardiopulmonary bypass is still the best method of treatment for the association between coronary fistula and severe coronary artery disease.

## Abbreviations

LAD: Left anterior descending
PA: Pulmonary artery.
